# VYC-25L Is Safe and Effective for Enhancing the Chin and Jawline by Correcting Chin Retrusion in Chinese Adults

**DOI:** 10.1093/asj/sjaf033

**Published:** 2025-04-17

**Authors:** Yun Xie, Qingfeng Li, Hongyi Zhao, Zuoliang Qi, Jiaming Sun, Qian Tan, Dong Li, Zhiqi Hu, Ziyang Liu, Smita Chawla

## Abstract

**Background:**

Individuals with chin retrusion may seek chin and lower jawline aesthetic enhancement.

**Objectives:**

To evaluate VYC-25L (Juvéderm Volux XC; Allergan Aesthetics, an AbbVie company), a lidocaine-containing injectable hyaluronic acid dermal filler, for chin retrusion correction.

**Methods:**

In this prospective, Phase 3, multicenter study (NCT04559984), Chinese adults with moderate-to-severe chin retrusion on the China (Allergan) Chin Retrusion Scale (CACRS) and glabella–subnasale–pogonion (G–Sn–Pog) angle <172.5° were randomized 2:1 to VYC-25L (treatment plus optional touch-up 4 weeks later) or no treatment (control). The primary endpoint was change from baseline (CFB) in G–Sn–Pog angle at Week 24. Secondary endpoints included CACRS response (≥1-point improvement), Global Aesthetic Improvement Scale (GAIS) response (improved/much improved), and FACE-Q Satisfaction with Chin score. Procedural pain was rated (0 = none to 10 = worst imaginable); injection-site responses (ISRs) were recorded daily for ≤28 days.

**Results:**

The mean age of the patient (VYC-25L, *n* = 97; control, *n* = 51) was 31.2 years (range, 20-52 years). Mean CFB in G–Sn–Pog angle at Week 24 was 2.97° (VYC-25L) vs 0.09° (control; between-group difference, 3.08° [*P* < .0001]); improvement was maintained to Week 52. At Week 24, VYC-25L achieved higher responses vs control for CACRS (78.7% vs 18.8%, respectively; rate difference, 60.0%; *P* < .0001) and GAIS (92.6% vs 4.2%, respectively; rate difference, 88.4%; *P* < .0001); mean overall FACE-Q Satisfaction with Chin score was 70.4 vs 34.9, respectively. Mean (standard deviation) procedural pain was 2.6 (1.6); most ISRs were mild (41.8%) or moderate (50.0%) in severity.

**Conclusions:**

In Chinese adults, VYC-25L safely and effectively corrected chin retrusion for at least 1 year.

**Level of Evidence: 2 (Therapeutic):**

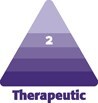

Chin shape and projection contribute to overall facial harmony and symmetry, and influence perceptions of attractiveness.^1,2^ Chin retrusion, or deficient projection of the chin,^1^ has been reported as undesirable by Chinese individuals, who generally prefer a pointy, well-projected chin as documented in a national online survey of mainland China residents^3^ and in consensus statements developed by Asian facial aesthetics experts.^2^ These cultural preferences may lead Chinese individuals to seek aesthetic enhancement of the chin and lower jawline to correct chin retrusion.^2^

Hyaluronic acid (HA) injectable fillers are commonly used for nonsurgical reshaping of the chin and jawline.^4,5^ VYC-25L (Juvéderm Volux XC; Allergan Aesthetics, an AbbVie company, Irvine, CA) is a lidocaine-containing injectable gel filler with a high concentration of HA (25 mg/mL) that allows for facial contouring and shaping.^6^ In a randomized clinical study in a European population of adults with chin retrusion, treatment with VYC-25L safety and effectively created volume in the chin and jawline, improved patients' satisfaction with their chin, lower face, and jawline appearance, and improved their psychological well-being.^7^ Similarly, a randomized clinical study enrolling US adults with loss of jawline definition demonstrated that treatment with VYC-25L improved jawline definition and patients' satisfaction with their lower face and jawline appearance.^8,9^ VYC-25L treatment has not been evaluated in a randomized clinical trial of Chinese adults with chin retrusion. Thus, the objective of the current study was to assess the safety and effectiveness of VYC-25L treatment to correct moderate-to-severe chin retrusion in adult Chinese patients seeking improvement of chin retrusion.

## METHODS

### Study Design

**Figure 1.**
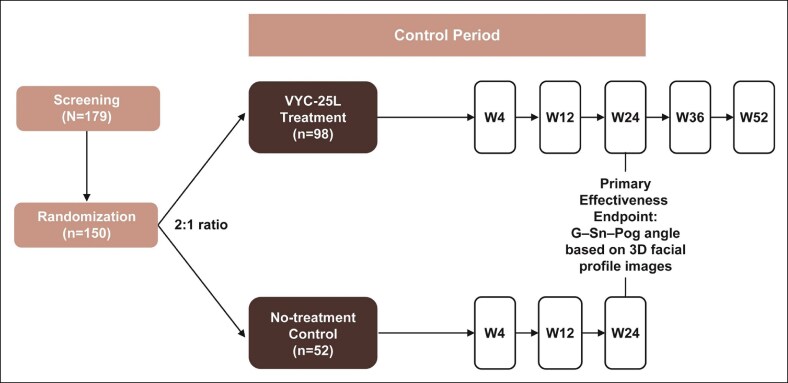
Study design. G–Sn–Pog, glabella–subnasale–pogonion; W, week.

**Figure 2.**
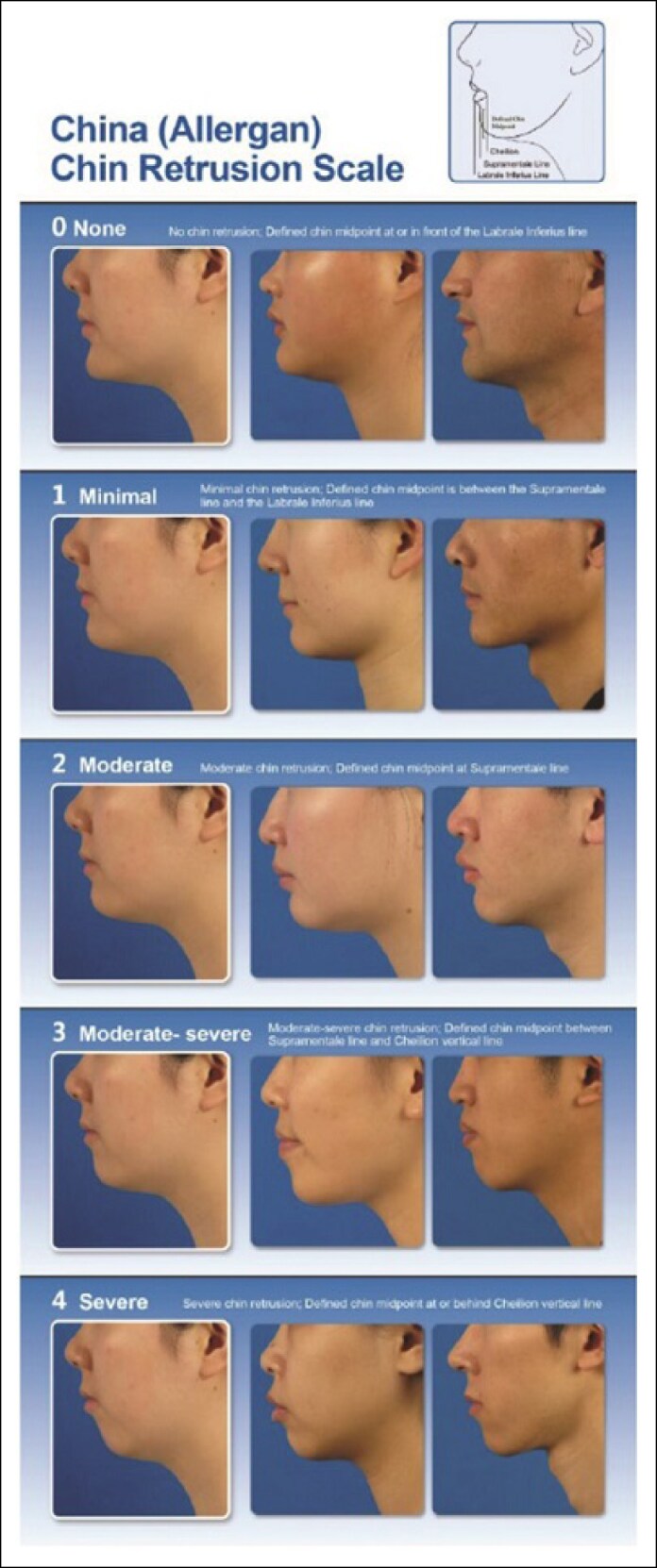
China (Allergan) Chin Retrusion Scale. © 2025 AbbVie. Used with permission.

This was a prospective, multicenter, Phase 3, randomized, no-treatment control study carried out at 7 sites in mainland China from October 2020 through August 2022 (NCT04559984). Patients were randomized in a 2:1 ratio to receive treatment with VYC-25L (treatment group) or no treatment (control group; Figure 1). Those randomized to the treatment group received initial treatment with VYC-25L on the same day as randomization and had follow-up visits at Weeks 4, 12, 24, 36, and 52 after randomization. Patients in the treatment group had the option to receive a touch-up treatment 4 weeks after the initial treatment if investigators determined that at least a 1-point improvement in the China (Allergan) Chin Retrusion Scale (CACRS; Figure 2) score had not been achieved. Patients randomized to the control group did not receive treatment during the first 24 weeks after randomization (control period). Follow-up visits in the control group occurred at Weeks 4, 12, and 24 after randomization. Control group patients could receive optional initial treatment at Week 24; however, those treatment outcomes are not presented here.

This study was approved by an independent ethics committee (Medical Ethics Committee of Shanghai Ninth People's Hospital, Shanghai Jiao Tong University School of Medicine, Shanghai, China) before initiation and was conducted in compliance with International Council on Harmonisation Good Clinical Practice guidelines, applicable regulations and guidelines for clinical study conduct, and ethical principles that have their origin in the Declaration of Helsinki. Patients provided written informed consent at screening, before the initiation of any procedures.

### Patients

Eligible patients were of Chinese descent, aged ≥18 years, who were seeking improvement of chin retrusion. Patients were required to have moderate-to-severe chin retrusion on the CACRS, as determined by the evaluating investigator assessment of 2-dimensional (2D) images; a reasonable goal for aesthetic improvement; and the ability to achieve a 1-point improvement in CACRS score with study treatment in the judgment of the treating investigator. Patients also were required to have a glabella–subnasale–pogonion (G–Sn–Pog) angle <172.5°, based on calculations of facial angle derived from digital images (Canfield Scientific, Parsippany, NJ). Individuals were excluded from enrollment if they had a history of a tendency to develop hypertrophic scarring; a history of anaphylaxis or allergy to lidocaine (or any amide-based anesthetics), HA products, or streptococcal protein; active autoimmune disease; current cutaneous inflammatory or infectious processes; previous chin or jaw surgery; permanent dermal filler injected below the subnasale, or semi-permanent dermal filler or fat injected below the subnasale within 36 months before enrollment. Also excluded were those who had a temporary dermal filler injected below the subnasale within 12 months before enrollment; had undergone orthodontics procedures within 12 months before enrollment; received botulinum toxin treatment in the chin area within 6 months before enrollment; or had undergone mesotherapy or cosmetic facial procedures (eg, facelift, brow lift, facial reconstructive surgery, laser, photomodulation, intense pulsed light, radiofrequency, dermabrasion, moderate or greater depth chemical peel, or other procedures) below the subnasale within 6 months before enrollment. Females who were pregnant, nursing, or planning a pregnancy during the study also were excluded.

### Treatment Administration

Treating investigators were experienced in the use of HA implants and were practicing physicians in the field of aesthetic medicine, plastic/cosmetic/reconstructive surgery, or dermatology. Before study initiation, each treating investigator received training in the administration of VYC-25L according to the injection technique specified for this study. The technique was designed to obtain optimal enhancement of the chin and mandible by correcting chin retrusion and achieving aesthetic improvement with minimal safety concerns.

VYC-25L was injected supraperiosteally with a 27-G, half-inch needle. Treatment was limited to the pogonion (a required initial treatment site), menton, and prejowl sulci. Anesthesia (topical or local injectable) was permitted at the treatment area per the treating investigator's standard practice. A minimum volume of 1.0 mL and a maximum volume of 4.0 mL were allowed for initial and touch-up treatments combined.

### Effectiveness Assessments

The primary effectiveness endpoint was change from baseline (CFB) in the G–Sn–Pog angle at Week 24. This angle was measured by a blinded image-analysis technician using digital images of the patient's facial profile. The blinded technician identified glabella, subnasale, and pogonion anatomical landmarks for each patient during the screening visit. On subsequent visits, the blinded technician aligned the glabella, subnasale, and pogonion landmarks based on the positions set in the digital images captured during screening. For patients with severe retrognathia, an automated script placed the pogonion landmark based on the initial landmark placement, which was adjusted by the blinded technician as needed. After the landmarks were positioned, the G–Sn–Pog angle was calculated by the program software using a built-in algorithm (Canfield Scientific).

Secondary effectiveness endpoints were assessed at Week 24 and included CACRS responder rate, investigator- and patient-assessed Global Aesthetic Improvement Scale (GAIS) responder rates, and patient-assessed FACE-Q Satisfaction with Chin overall score. The CACRS is a validated photonumeric scale for assessing chin retrusion in Chinese patients on a scale of 0 (none) to 4 (severe) based on 2D images.^10^ A CACRS responder was defined as a patient who had ≥1-point improvement from baseline in CACRS score. The GAIS is a 5-point scale on which patients (VYC-25L treatment group only) and evaluating investigators rated global aesthetic improvement of the chin and jaw area from −2 (much worse) to 2 (much improved) compared with profile view photographs taken at baseline. Patients used a mirror to assess their current appearance at each visit. A GAIS responder was defined as a patient whose chin and jaw area was rated as improved (score of 1) or much improved (score of 2). The FACE-Q Satisfaction with Chin is a 10-item questionnaire assessing patient satisfaction with different aspects of the chin (eg, size, width, and shape of the chin, how chin looks in profile) on a 4-point scale from very dissatisfied to very satisfied. The sum of the items was calculated and converted to Rasch-transformed scores ranging from 0 (worst) to 100 (best).

Other effectiveness endpoints were CFB on the FACE-Q Satisfaction with Lower Face and Jawline scale, a 6-item questionnaire assessing patient satisfaction with various aspects of their lower face and jawline (eg, jawline prominence and definition, how jawline looks in profile; 4-point scale from very dissatisfied to very satisfied); CFB on the FACE-Q Psychological Function scale, a 10-item questionnaire assessing how facial appearance impacts a patient's self-image (eg, feels happy, confident, attractive, feels comfortable, and positive about myself; 4-point scale from definitely disagree to definitely agree); and CFB in chin area volume change, as calculated by a blinded image analysis technician from baseline and follow-up 3-dimensional images.

### Safety Assessments

Procedural pain was assessed by patients on an 11-point scale (0 = no pain to 10 = worst pain imaginable) immediately after each treatment (initial and touch-up). The presence and severity of injection-site responses (ISRs) were assessed and recorded in daily diaries by patients for up to 28 days after each treatment (initial and touch-up), starting on the day of treatment. Any ISRs that were ongoing at the end of the diary (Day 28) were reported on the ongoing ISR eCRF and tracked until resolution or until follow-up was no longer possible. Diaries listed the following ISRs, which had been reported previously with HA dermal injections: redness, pain after injection, tenderness to touch, firmness, swelling, lumps/bumps, bruising, itching, and discoloration. Severity was rated as none, mild, moderate, or severe. Treatment-emergent adverse events (TEAEs) were monitored throughout the study.

### Statistical Analysis

A total of 126 patients were needed to provide >97% power to detect a difference in mean G–Sn–Pog angle CFB of at least 1.6° between groups and a common standard deviation (SD) of 2.1, based on a 2-sided, 2-sample *t* test at the 5% level. Therefore, 150 patients were enrolled, of which ∼126 patients (84 in the treatment group and 42 in the control group) were expected to complete Week 24, assuming a dropout rate of 15% before Week 24.

Effectiveness analyses were performed on the modified intent-to-treat (mITT) population, defined as all randomized patients who had ≥1 postbaseline assessment of the G–Sn–Pog angle. The safety analysis was performed on the safety population, defined as all randomized patients who received study treatment. Descriptive statistics were performed; all statistical tests were 2-sided hypothesis tests performed at the 5% level of significance and performed using SAS version 9.4 or later (Cary, NC).

## RESULTS

### Patients

Of 179 individuals screened, 150 were randomized and 148 comprised the mITT population. The mean age of the mITT population was 31.2 years (SD, 7.6; range, 20-52 years); 17 patients (11.5%) were males and 131 patients (88.5%) were females. During the control period, 8 patients discontinued the study because of withdrawal by patient (VYC-25L group, *n* = 2; control group, *n* = 4), protocol deviation (VYC-25L group, *n* = 1), and lost to follow-up (VYC-25L group, *n* = 1). Patient baseline characteristics are summarized in Table 1.

**Table 1.** Patient Demographic and Baseline Clinical Characteristics (mITT Population)

Characteristic	VYC-25L treatment group *n* = 97	Control group *n* = 51	Total population *n* = 148
Age, years			
Mean (SD)	31.4 (7.6)	30.9 (7.7)	31.2 (7.6)
Range	21-52	20-50	20-52
Sex, *n* (%)			
Male	12 (12.4)	5 (9.8)	17 (11.5)
Female	85 (87.6)	46 (90.2)	131 (88.5)
BMI, kg/m^2^			
Mean (SD)	21.3 (2.8)	20.9 (2.4)	21.1 (2.7)
Range	15.1-28.7	17.1-28.1	15.1-28.7
G–Sn–Pog angle, degrees			
Mean (SD)	162.1 (4.5)	162.0 (5.4)	NR
Range	151.6-171.5	149.0-172.3	149.0-172.3
FACE-Q Satisfaction with Chin Scale, mean (SD) transformed score	32.1 (11.2)	30.8 (9.8)	NR

G–Sn–Pog, glabella–subnasale–pogonion; mITT, modified intent to treat; NR, not reported; SD, standard deviation.

### Treatment Administration

All patients in the treatment group received initial treatment with VYC-25L in the pogonion with a majority also receiving treatment in the menton, right prejowl sulcus, and/or left prejowl sulcus. The mean (SD) volume of VYC-25L injected across all areas was 2.4 (0.85) mL and anesthesia (ice) was used for 70 (71.4%) patients. A total of 19.4% (*n* = 19/98) of patients in the treatment group received VYC-25L touch-up treatment, which was in line with the rate of CACRS nonresponders at Week 4 (18.8%). Of them, 47.4% received touch-up treatment in the pogonion, and 52.6%, 73.7%, and 89.5% received touch-up treatment in the menton, right prejowl sulcus, and left prejowl sulcus, respectively. The mean (SD) volume of VYC-25L injected was 1.2 (0.58) mL, with 12 of 19 (63.2%) patients using anesthesia (ice). At Week 24, 42 patients in the control group (80.8%) chose to receive an optional VYC-25L treatment, of which 8 patients (19.0%) received a subsequent touch-up.

### Effectiveness Endpoints

**Figure 3.**
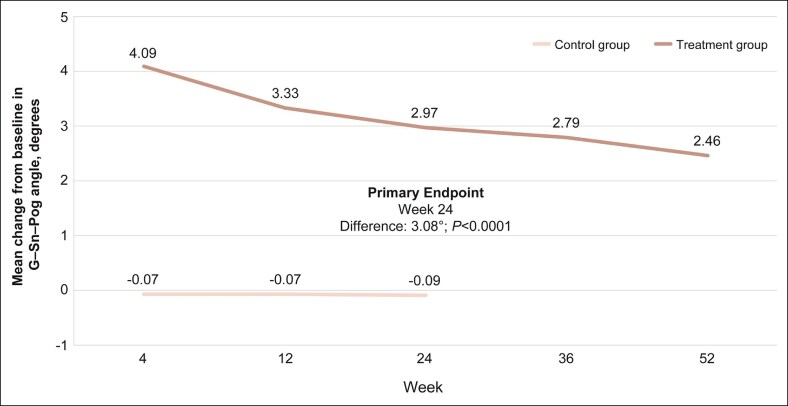
Mean change from baseline in G–Sn–Pog angle over time through Week 52. G-Sn-Pog, glabella–subnasale–pogonion; W, week.

The primary effectiveness endpoint was achieved (Figure 3). The mean CFB in the G–Sn–Pog angle was significantly larger in the VYC-25L treatment group compared with control at all time points, including Week 24 (2.97° vs −0.09°, respectively), with a between-group difference of 3.08° (*P* < .0001). The mean CFB in the G–Sn–Pog angle in the VYC-25L group was maintained at 2.79° and 2.46° through Weeks 36 and 52, respectively.

**Figure 4.**
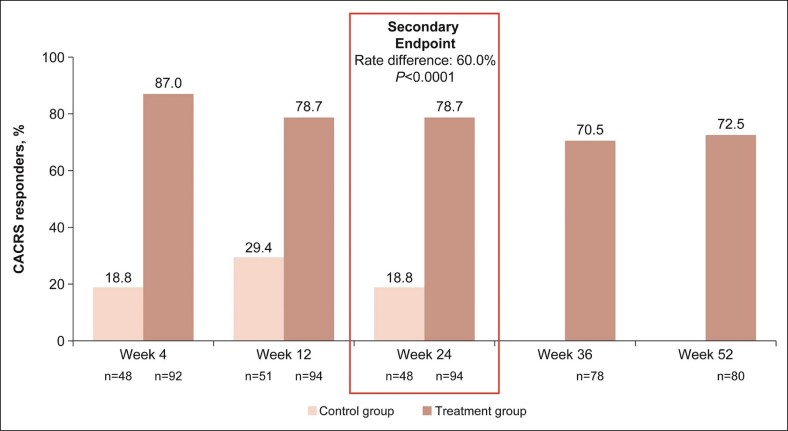
China Allergan Chin Retrusion Scale responder rates at all time points. CACRS, China Allergan Chin Retrusion Scale.

The CACRS responder rate (Figure 4) at Week 24 was significantly higher in the VYC-25L group than in the control group (78.7% vs 18.8%, respectively; rate difference, 60.0%; *P* < .0001). CACRS responder rates at Weeks 4 and 12 were higher in the VYC-25L group vs control and remained high through Week 52.

**Figure 5.**
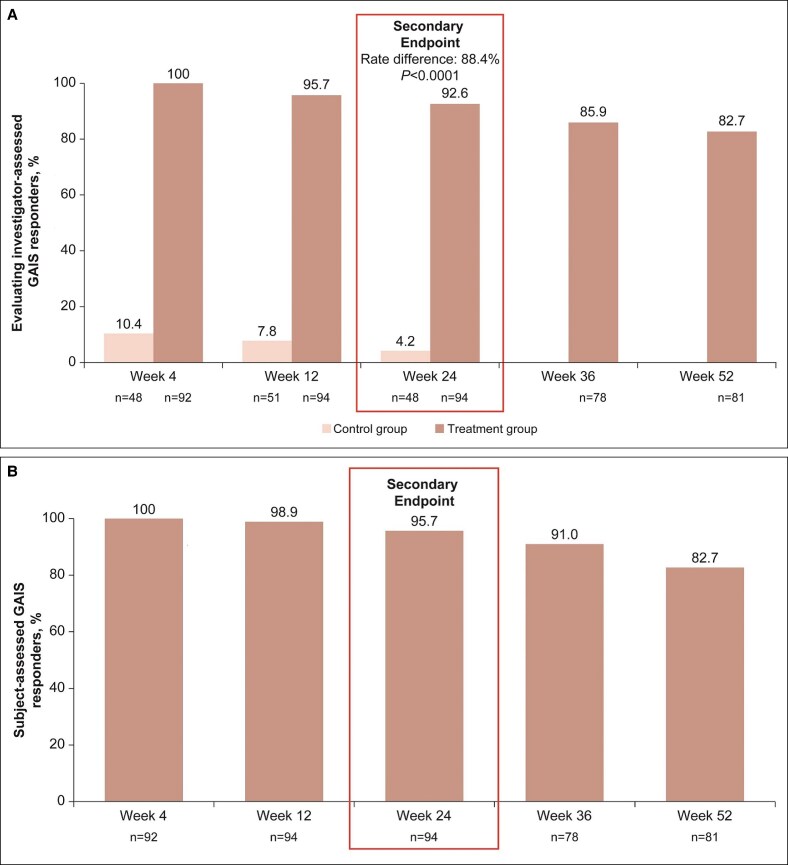
GAIS responder rates at all time points. (A) Evaluating investigator-assessed GAIS responder rates. (B) Patient-assessed GAIS responder rates. GAIS, Global Aesthetic Improvement Scale.

The investigator-assessed GAIS responder rate at Week 24 was higher in the VYC-25L group vs control (92.6% vs 4.2%, respectively), for a significant between-group rate difference of 88.4% (*P* < .0001; Figure 5A). The patient-assessed GAIS responder rate at Week 24 was 95.7% in the VYC-25L group (Figure 5B), similar to that of the evaluating investigators. Both investigator- and patient-assessed GAIS responder rates remained high in the VYC-25L group through Week 52.

**Figure 6.**
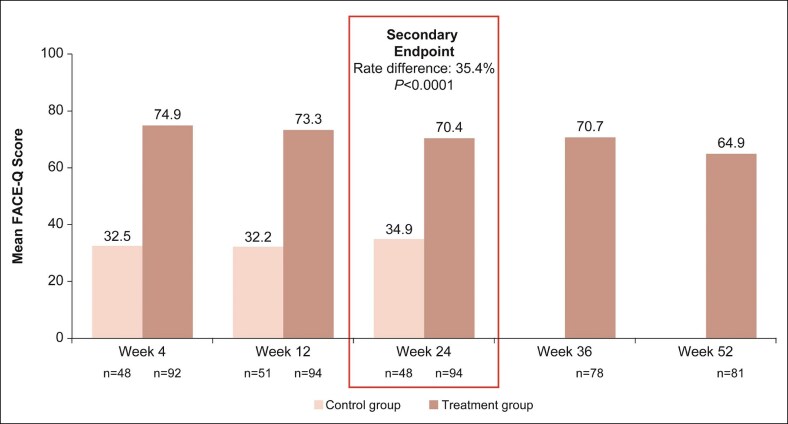
FACE-Q Satisfaction with Chin Scale scores at all time points.

The mean overall FACE-Q Satisfaction with Chin score (Figure 6) at Week 24 was higher in the VYC-25L group than in the control group (70.4 vs 34.9, respectively), representing a mean CFB of 37.8 in the VYC-25L group and 3.5 in the control group, and a significant between-group rate difference of 35.4 (*P* < .0001). Mean overall scores in the VYC-25L group remained high through Week 52.

Mean overall CFB in FACE-Q Satisfaction with Lower Face and Jawline score was increased in the VYC-25L group and remained generally unchanged in the control group at Weeks 4, 12, and 24. At Week 24, the mean (SD) CFB was 44.1 (24.6) in the VYC-25L group and 4.2 (12.1) in the control group. At Weeks 4, 12, and 24, most patients in the control group were somewhat/very dissatisfied with the appearance of their lower face and jawline (Week 24, range, 79.2%-93.8%), whereas most patients in the VYC-25L group were somewhat/very satisfied with the appearance of their lower face and jawline (Week 24, range, 78.7%-80.9%). In the VYC-25L group, mean increases from baseline in FACE-Q Satisfaction with Lower Face and Jawline score persisted through Week 52.

Mean overall FACE-Q Psychological Function scores increased from baseline in the VYC-25L group and decreased from baseline in the control group at Weeks 4, 12, and 24. At Week 24, the mean (SD) CFB was 12.6 (20.3) and −5.6 (17.4) in the VYC-25L vs control group, respectively. The overall FACE-Q Psychological Function score remained increased from baseline through Week 52 in the VYC-25L group.

At Week 24, the mean (SD) CFB in chin area volume was 4.2 (2.4) mL for the VYC-25L group vs 0.4 (1.7) mL for the control group. Chin area volume in the VYC-25L group increased from baseline at all time points, with median changes ranging from 4.0 to 5.2 mL; in the control group, chin area volume changes were minimal, ranging from 0.1 to 0.4 mL. Increase in chin area volume was maintained in the VYC-25L group through Week 52 (3.8 and 3.6 mL at Weeks 36 and 52, respectively).

**Figure 7.**
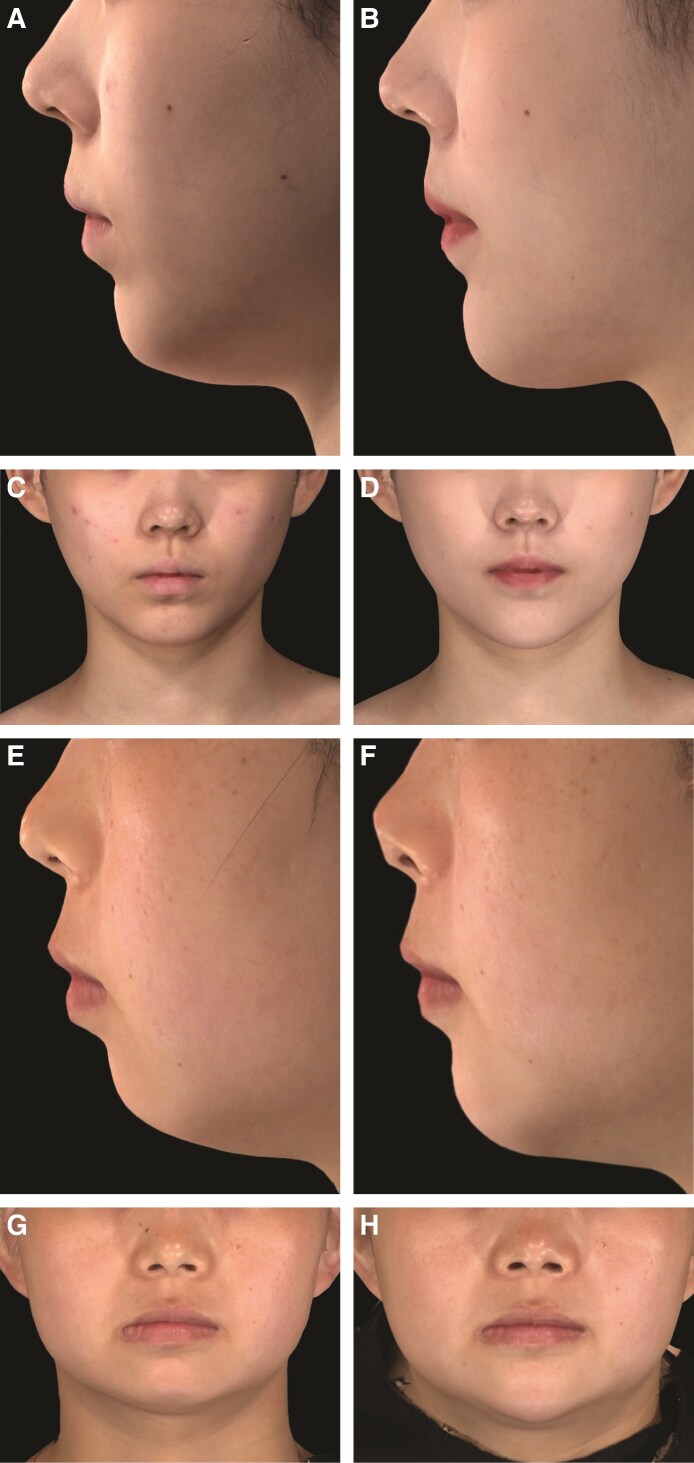

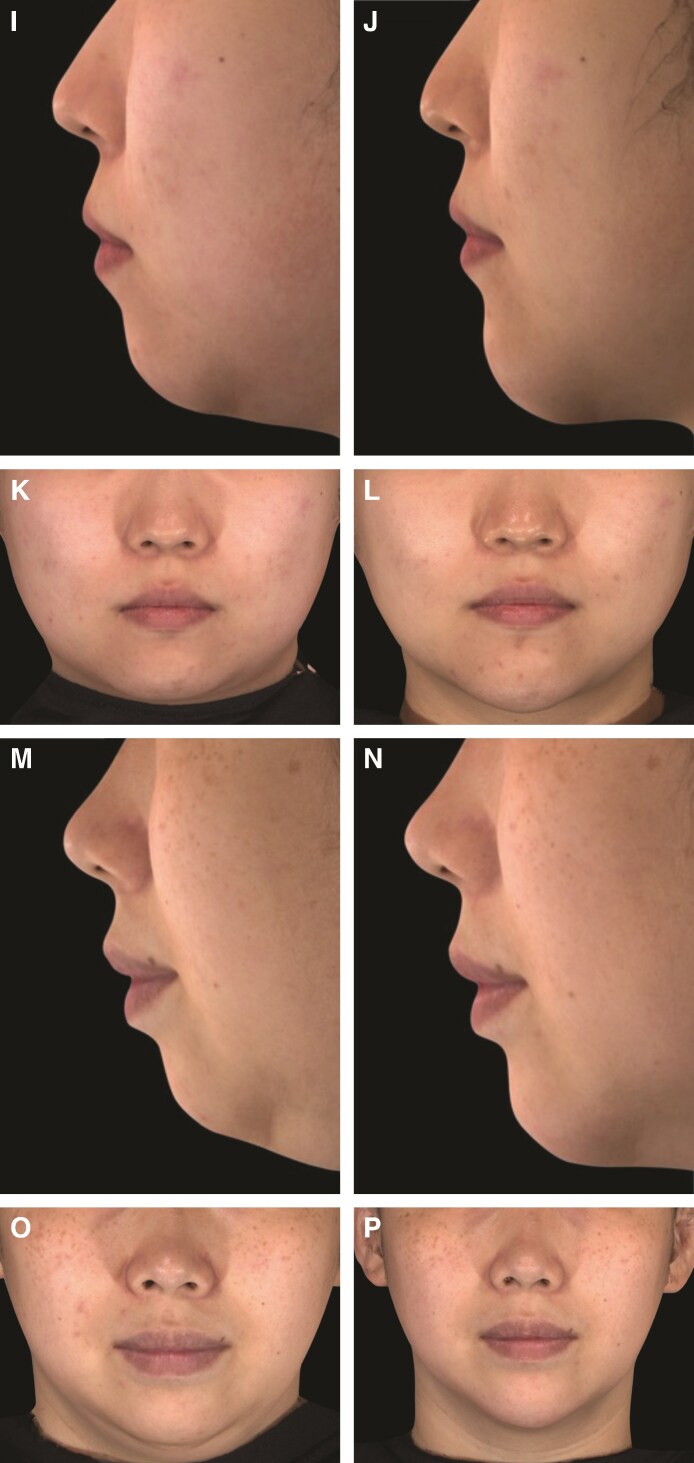
Representative photographs of patients before and after receiving VYC-25L showing improvements in the G–Sn–Pog angle. (A and C) Participant A (female aged 21 years) at baseline. CACRS score: 4; G–Sn–Pog angle: 165°. (B and D) Participant A (female aged 21 years) at Week 24 posttreatment. Total injected volume: 1.65 mL; CACRS score: 1; G–Sn–Pog angle: 169°; angle change from baseline: 4°. (E and G) Participant B (female aged 29 years) at baseline. CACRS score: 3; G–Sn–Pog angle: 165°. (F and H) Participant B (female aged 29 years) at Week 24 posttreatment. Total injected volume: 2 mL; CACRS score: 1; G–Sn–Pog angle: 169°; angle change from baseline: 4°. (I and K) Participant C (female aged 30 years) at baseline. CACRS score: 3; G–Sn–Pog angle: 166°. (J and L) Participant C (female aged 30 years) at Week 24 posttreatment. Total injected volume: 3.1 mL; CACRS score: 1; G–Sn–Pog angle: 169°; angle change from baseline: 3°. (M and O) Participant D (female aged 37 years) at baseline. CACRS score: 3; G–Sn–Pog angle: 159°. (N and P) Participant D (female aged 37 years) at Week 24 posttreatment. Total injected volume: 3.5 mL; CACRS score: 2; G–Sn–Pog angle: 162°; angle change from baseline: 3°. CACRS, China Allergan Chin Retrusion Scale; G-Sn-Pog, glabella–subnasale–pogonion.

Representative photographs of patients at baseline and 24 weeks after initial treatment with VYC-25L, demonstrating improvement in the appearance of the chin and lower jawline, are shown in Figure 7.

### Safety

During the control period, 15 (15.3%) patients in the VYC-25L group and 3 (5.8%) patients in the control group experienced TEAEs. One TEAE in the VYC-25L group, hypoglycemia (1 event), was considered treatment-related. One patient in each group experienced a treatment-emergent serious AE (VYC-25L group, pneumonia [1 event]; control group, gastric adenoma [1 event]); none of the events were assessed as treatment-related. No TEAE led to study discontinuation. An overall summary of TEAEs is shown in Table 2.

**Table 2.** Summary of TEAEs Reported During the Control Period (Safety Population)

	VYC-25L treatment group		Control group	
	Patients, *n* (%), *n* = 98	Events, *n*	Patients, *n* (%), *n* = 52	Events, *n*
All TEAEs	15 (15.3)	30	3 (5.8)	4
TRAEs	1 (1.0)	1	0	0
At injection site	0	0	0	0
Not at injection stie	1 (1.0)	1	0	0
All SAEs	1 (1.0)	1	1 (1.9)	1
Treatment-related SAEs	0	0	0	0
TEAEs leading to study discontinuation	0	0	0	0
Deaths	0	0	0	0

SAEs, serious adverse events; TEAEs, treatment-emergent adverse events; TRAEs, treatment-related adverse events.

**Figure 8.**
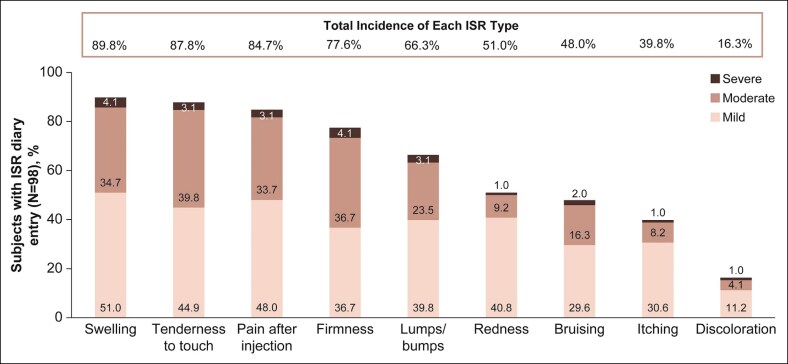
Injection-site responses after initial treatment. ISR, injection-site response.

All patients in the VYC-25L group reported ≥1 ISR after initial treatment, with the most common being swelling (89.8%), tenderness to touch (87.8%), and pain after injection (84.7%; Figure 8). Most ISRs reported at initial treatment were mild (41.8%) or moderate (50.0%) in severity; 8.2% were severe. Severe ISRs were firmness and swelling (each *n* = 4; 4.1%), tenderness to touch, pain after injection, and lumps/bumps (each *n* = 3; 3.1%), bruising (*n* = 2; 2.0%), and redness, itching, and discoloration (each *n* = 1; 1.0%). The majority of ISRs (67.4%) reported at initial treatment resolved within 14 days. The most common ISRs that lasted beyond 14 days were firmness (21.4%), tenderness to touch (11.2%), lumps/bumps (8.2%), and swelling (8.2%). Ongoing ISRs were reported in 12.2% of patients after initial treatment and in no patients after touch-up treatment, and included firmness (8.2%, mean [SD] duration = 48.0 [19.8] days), lumps/bumps (4.1%, mean [SD] duration = 41.0 [20.2] days), and tenderness to touch (2.0%, mean [SD] duration = 54.0 [28.3] days). No ISRs were reported as adverse events or inflammatory nodules. Mean (SD) procedural pain was rated as 2.6 (1.6) by patients (*n* = 98) in the VYC-25L group immediately after receiving initial treatment and 1.9 (1.3) by patients (*n* = 19) immediately after receiving VYC-25L touch-up treatment.

## DISCUSSION

In this study, VYC-25L was effective and well tolerated for the treatment of moderate-to-severe chin retrusion in Chinese individuals. Treatment with VYC-25L corrected the volume deficit in the chin and lower jaw, as reflected by significant improvements vs control in the G–Sn–Pog angle, and investigator-assessed CACRS as well as high patient-assessed GAIS responder rates, and FACE-Q measures of satisfaction. Posttreatment procedural pain was minimal, ISRs were mostly mild to moderate in severity and transient in nature, and no patient discontinued because of TEAEs. Improvements obtained in individuals with severe chin retrusion provide support for the approach of using VYC-25L as an alternative to surgery.

These results are consistent with findings of a real-world experience study of VYC-25L treatment for chin enhancement conducted at Hainan Bo’ao Super Hospital in China.^11^ In that study, VYC-25L was injected in the subcutaneous and/or supraperiosteal planes of the chin area and prejowl sulci. The mean CFB in the G–Sn–Pog angle 3 months after treatment (primary endpoint) showed improvement similar to that seen at the 12-week (3-month) time point in the current study. Similarly, as in the current study, improvements in the G–Sn–Pog angle and in the appearance of the chin and jawline were maintained through 1 year, patient satisfaction with treatment was high, and the majority of TEAEs were mild or moderate in severity.

Our results compare favorably with those of a randomized, evaluator-blinded, no-treatment control study of HA-DEF (Restylane Defyne, Galderma, Uppsala, Sweden), a lidocaine-containing HA filler, in Chinese adults with mild-to-moderate chin retrusion.^12^ Unlike that study, the present study evaluated VYC-25L effectiveness in patients with moderate-to-severe chin retrusion using objective assessments in addition to investigator- and patient-assessed subjective measures. Furthermore, chin retrusion at baseline and posttreatment in the current study was assessed using the CACRS, a validated scale developed specifically for Chinese adults who, compared with Western patients, have distinct craniofacial morphological characteristics, have different aesthetic goals, and more frequently present with lip protrusion and chin retrusion.^10^ In contrast, the scale used in the study of HA-DEF was not specific for Chinese adults.^12^

This study has some limitations. Most patients (88.5%) were females; thus, because the results may not be generalizable to males, our study population is representative of a real-world population of individuals who undergo filler injections, as demonstrated by recent data that males receive 6% to 9.7% of all minimally invasive cosmetic procedures and 4.1% to 8.6% of all filler procedures.^13,14^ In addition, the mean age of the study population was 31 years, which is younger than that in the European and US pivotal studies (46 and 59 years, respectively), limiting the generalizability of the results across age ranges. However, the mean age of the study population was similar to that of the population analyzed in the real-world experience study in China described above (33.2 years).^11^ Finally, the current study did not contain an active comparator; patients who served as controls received no treatment in the 24-week control period.

## CONCLUSIONS

VYC-25L had an acceptable safety profile and corrected moderate-to-severe chin retrusion in Chinese adults, with improvements observed through 1 year. In addition, treatment with VYC-25L was associated with high patient satisfaction. Thus, altogether, the results of this study suggest that VYC-25L is safe and effective in the enhancement of the chin and jaw area to restore volume in Chinese adults with chin retrusion.
